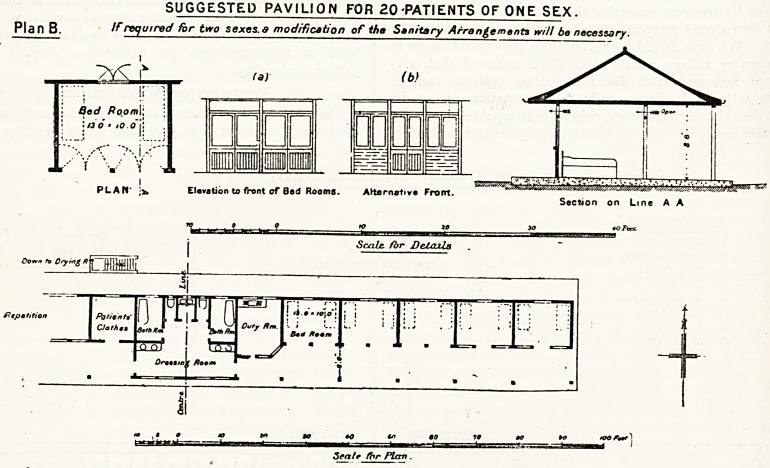# Tuberculosis Hospital & Sanatorium Construction

**Published:** 1913-03-15

**Authors:** 


### TUBERCULOSIS HOSPITAL & SANATORIUM CONSTRUCTION.

### Inexpensive Buildings for Tuberculosis Patients.

IN 1911 the United States, through the National Association for the Study and Prevention of Tuberculosis, for the second time issued an important brochure dealing with the problem of how to provide for the largest possible number of tuberculosis patients at the least possible cost compatible with efficient results. They insisted properly that careful preliminary planning can immensely reduce the original cost of construction. It is, indeed, the chief factor in subsequent economy of administration. This American publication is full of plans and information of very real value to rural communities especially. It shows by concrete examples that the cost of construction can be reduced comparatively to a relatively unimportant sum. We have found by an absolute experiment made in a country village where tuberculosis occurred that adequate accommodation for one or two patients can be constantly maintained ready for occupation at an inappreciable cost in money, whilst it is capable of expeditiously restoring cases in the early stages to sound health.

Plan A

SUGGESTED PAVILION FOR 50 PATIENTS (25 OF EACH SEX)

A letter we published last week insisted upon Government action, failing the exhibition on the part of British architects of the will and power to plan and erect inexpensive buildings for tuberculosis. We welcome, therefore, the important Circular and Memorandum just issued by the Local Government Board. We referred to these documents last week, but in view of their importance we publish to-day the full Memorandum, signed by the Architect and Medical Officer to the Board in testimony of their present solid and useful practical work. The Local Government Board properly urges upon the County Councils and County Borough Councils the importance of organising without delay an efficient system of dispensaries in their area. These dispensaries can treat a large proportion of cases and form centres where suitable cases for treatment in residential institutions can be most satisfactorily selected so as to secure the immediate treatment of patients in the first stage of the disease in separate sanatoria, which are essential to successful treatment. Sir H. C. Monro, Secretary to the Board, points out that the capital grant made to aid the provision of santoria is a fixed sum, a fact which must govern the decision to make grants. It follows that applications for funds should not be delayed.

### The Construction and Arrangement of Inexpensive Buildings.

By BROOK KITCHIN and ARTHUR NEWSHOLME, M.D., F.R.C.P.

The object of this Memorandum is to indicate in a general way the manner in which sanatorium accommodation of an inexpensive character may be erected within a comparatively short period in order to meet any pressing need of accommodation for tuberculous persons in a particular locality.

PROVISION OF ACCOMMODATION.

The manner in which such accommodation may be most easily and economically provided is by erecting such buildings as are hereafter described, either as a self-contained sanatorium or an independent site, or as an adjunct to an existing institution belonging to a local authority, whether for tuberculosis or for acute infectious diseases.

FORMS OF CONSTRUCTION.

Accommodation for patients may be readily built at a comparatively small cost of timber framing, weather boarded and creosoted, or covered with corrugated iron or other suitable material, such as tile hanging, asbestic sheeting, or covered with expanded metal and cemented, and covered internally with lath and plaster, asbestic sheeting, etc.; and a portion of this accommodation may be temporarily arranged and used for the accommodation of the additional staff until complete and permanent quarters have been provided.

In addition to the above method, the following forms of construction, which do not, however, lend themselves to such rapid and economical construction, but are of a more permanent character, may be employed: (a) Buildings constructed of steel framing filled in with terra-cotta or concrete slabs, (*b*) hollow concrete blocks, (*c*) solid concrete. These forms of construction would, under favourable circumstances, be somewhat cheaper than brickwork, but their economy depends upon the ready access to suitable materials and upon the presence of labour skilled in such special forms of construction; further, it must be borne in mind that risks of settlement and fracture are incidental to buildings of concrete which have been hurriedly and perhaps carelessly constructed.

### Relative Cost of Walls.

The relative initial cost of the walling constructed of these various forms in comparison with walls of 9-in. brickwork may, under ordinary conditions, be approximately reckoned as follows :—

Walls of timber framing covered with weather-boarding or expanded metal lathing rendered in cement and plastered internally, from 25 to 30 per cent. cheaper.

Walls constructed of concrete blocks or solid concrete, 20 per cent. cheaper.

Walls constructed with terra-cotta or concrete slabs, 15 to 20 per cent. cheaper.

### Timber-framed Buildings.

Under ordinary conditions, the cost of patients' accommodation constructed of timber framing should not exceed £5 per bed, exclusive of administrative department, etc.

Timber-framed buildings, though less permanent and requiring greater cost in upkeep, have the advantage of requiring little or no foundations. They may be erected on a concrete platform, which may also form the flooring of the wards and verandahs. The actual amount of brickwork required will be almost negligible. Where sufficient labour for the construction of such buildings cannot readily be obtained, it may be necessary to have recourse to firms who make a speciality of the rapid erection of buildings, and who keep in stock the materials ready for emergencies.

### Types of Buildings.

The type of building which is most suitable for the purpose will vary according to the particular needs and circumstances of the locality in which it is to be erected and for which it is required. The accompanying diagram plans are intended merely to illustrate two types which would generally be suitable :—

Plan A is a pavilion for fifty beds (twenty-five of each sex) which might form an adjunct to an existing hospital or form the basis of an independent sanatorium with an administrative department, which is shown approximately by dotted lines on the plan. It is suggested that such a building might be constructed of timber framing on a concrete platform, and that it should comprise a series of two bed wards each 13 ft. by 10 ft., and one ward for twelve beds, with a central dining-hall large enough to accommodate, besides the patients in the pavilion itself, any patients that might at a future date be accommodated in other pavilions or châlets. The room shown on the plan would be capable of dining 100 persons.

**SUGGESTED PAVILION FOR 20 PATIENTS OF ONE SEX.**

**Plan B.** *If required for two sexes a modification of the Sanitary Arrangements will be necessary.*

Plan B is a design for a pavilion of similar construction to contain twenty beds of one sex, intended to supplement existing accommodation. If a still smaller number of additional beds will suffice, the block can be reduced according to requirements.

### Details.

The divisions between the bedrooms may be formed of timber framing covered with expanded metal or wood lathing and plastered, or of coke breeze or cement slabs two or three inches thick. The front wall of the patients' bedrooms should be framed in timber and fitted with two pairs of French casement doors, each door being hung in two sections, on the "stable door" pattern, or, as an alternative, with one pair of doors having window openings on each side, which may be left open and fitted with roller blinds, or may be fitted with casement windows opening the full extent of the window-frame (see detail *b*). The space above the door and window-frame, and a corresponding space on the back wall, should be left completely open for ventilation (see section). If the local circumstances are suitable, the front of each bedroom or of some of the bedrooms may be left entirely open.

The floors of the wards may be finished in cement and covered with linoleum or covered with narrow floor boards laid in pitch. The verandah floors should be of granolithic finish, with a slight fall to the outside.

In the event of temporary accommodation for the nurses being required pending the completion or erection of administrative buildings, some of the bedrooms might be specially arranged for their use in such a way that they can readily be adapted later for the use of patients.

Hot water can be supplied from a boiler in a basement under one of the duty rooms, while in a corresponding basement on the other side a boiler for heating the dining-hall, etc., could be installed. These rooms may also be used as drying-rooms for patients' clothes, bedding, etc.

Provision should be made for the satisfactory disinfection and disposal of sputum and for the cleansing of sputum cups after disinfection, preferably by steam.

The nurses' duty rooms should be fitted with sink and with gas range or small cottage coal range, which may be of the "portable" type, and a small ventilated food store.

Sleeping shelters and châlets, if provided, should form only a small proportion of the total accommodation, in view of administrative difficulties. Where the special circumstances of the case justify this form of accommodation on a limited scale, they should be erected on concrete foundations, and provision made for adequate supervision and for convenient access of the patients to sanitary conveniences, etc.

Where a drainage system is not already in existence, some arrangements will be necessary for purifying the sewage before it is discharged into a stream or watercourse. Where suitable and sufficient land is available, purification by land treatment will be the most satisfactory. Earth closets are only permissible under exceptional circumstances or as a temporary arrangement. If the use of earth closets should be necessary, they should be isolated from the building containing the patients' accommodation.

Arthur Newsholme,
Medical Officer to the Board.
Brook Kitchin,
Architect to the Board.

## Figures and Tables

**Plan A f1:**
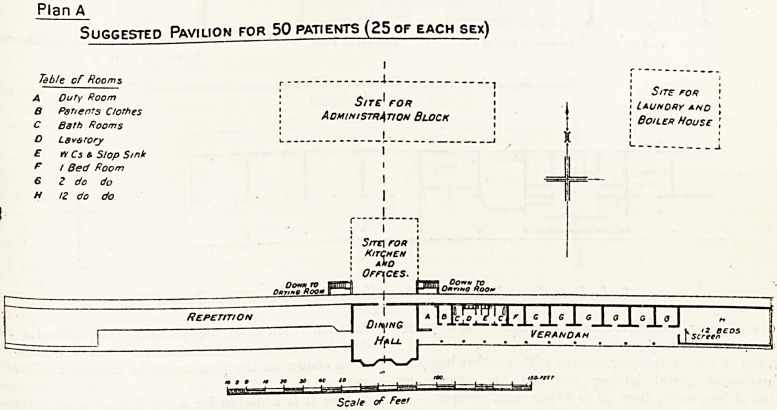


**Plan B f2:**